# The Impact of Water Sporting Events on Attitudes Toward Physical Activity: Motivational Profiles of Participants in Modern and Traditional Water Events

**DOI:** 10.3389/fpsyg.2021.632948

**Published:** 2021-03-30

**Authors:** Maciej Młodzik, Marek Kazimierczak, Patxi León-Guereño, Miguel Angel Tapia-Serrano, Ewa Malchrowicz-Mośko

**Affiliations:** ^1^Eugeniusz Piasecki Academy of Physical Education, Institute of Sport Sciences, Poznań, Poland; ^2^Department of Psychology and Education, University of Deusto, San Sebastian, Spain; ^3^Department of Didactics of Musical, Plastic and Body Expression, Faculty of Teaching Training, University of Extremadura, Cáceres, Spain

**Keywords:** water sports, sport management, modern sporting events, traditional sporting events, health promotion, event management, cultural diffusion and sport

## Abstract

The aim of this paper was to analyze the relationship between attitudes toward physical activity and participation in water sports events and to recognize the main motives for involvement in these kinds of events. A written paper–pencil diagnostic survey was conducted among 394 participants in two traditional and two modern sports events on water held in Poland to ascertain whether innovative (modern) events are needed in society, and whether they cause an increase in interest in physical activity (more than traditional events). The research results showed that modern sports events on water did not have any more power to attract physically inactive people than traditional water events, did not produce a greater desire to lead an active lifestyle, and did not encourage people to more regularly practice water sports. Moreover, modern events, compared to traditional events, were only a one-time experience and the people who completed survey often wanted to return to traditional sports events which provided participants with greater positive emotions than a modern event. In the case of socio-demographical variables, there was no statistically significant relationship between gender and choice of modern or traditional event, but older people were more likely to choose a traditional event than younger people; modern water events were an attractive option primarily for young people under 30 years of age, furthermore, modern events more often attracted people who had completed higher education. However, it turned out that a modern event on water often attracted more people who had had no experience in this sport discipline rather than people who chose traditional water events. In a sense, modern events are therefore effective in promoting water sports in Polish society. We also distinguished five main groups of participants: healthy lifestyle managers, lovers of sports emotions, water sports malcontents, water sports enthusiasts, and neutrals to water sports. Additionally, we looked into gender-related motives for participation in modern and traditional water events: social and health-related motives proved to be more important for women and men who participated in modern water events. The research results presented in the article expand on the current state of knowledge about mass participation in sport, the impact of sporting events on the promotion of physical activity, and show the motivation behind participation in modern and traditional water sporting events.

## Introduction

There is a widespread belief that the organization of non-elite mass sports events has the potential to promote physical activity – sporting events provide strong emotions, long-lasting social relationships, and give a chance for amateurs, who are strong motivators of modern sport, to also compete, which can encourage regular physical activity after the event (Bennett et al., 2007; Murphy and Bauman, 2007; Bauman et al., 2009; Funk et al., 2011; Crofts et al., 2012; Stevinson and Hickson, 2014; Coleman and Sebire, 2016). However, little is known about the real effectiveness of such events in promoting health. In practice, the impact of organizing sporting events on the promotion of physical activity, a healthy lifestyle, and participation in sport in scientific literature is debatable. In addition, most of the scientific literature in this field concerns elite mega-sports events such as the Olympic Games or world championships (Frawley and Cush, 2011). This raises the question as to the impact of medium- and small-scale sporting events, including mass sports events. Research on the effects of events, the so-called sports legacy, often refers to tangible effects, such as infrastructure left after a competition (sports, recreation, or transport infrastructure) or the purchase of specialist sports equipment (Mcgillivray et al., 2015; Misener, 2015). However, the construction of new sports and recreation facilities does not automatically attract their users. Despite the high expectations of event organizers regarding their impact on people's health behavior, few studies provide empirical evidence to suggest that events encourage people to be more physically active. This article focuses on water sports because previous studies on active participation in mass sports events relate to other, more popular activities, i.e., running or cycling (Malchrowicz-Mośko and Poczta, 2018; Malchrowicz-Mośko et al., 2019a,b, 2020a). Buning and Walker (2016) analyzed the motivation behind participants of traditional and non-traditional running events using a runner motivation questionnaire, MOMS (Motivation of Marathoners Scale) (Buning and Walker, 2016). However, an event such as a marathon is different, for which you need to make solid preparations, while the water sports and recreational events analyzed in this paper can include people without extensive sports experience and psychophysical preparation. Nowadays, event organizers are trying to highlight something that stands out against the backcloth of organized competitions, there are many new sports disciplines, and those that already exist are subject to further diversification. Modern sporting events, previously unknown in a given cultural circle, are now appearing in the leisure industry. In order to attract the younger generation, for example, in extreme sports, such disciplines as horse boarding contests have emerged in Poland (snowboarding while attached to a horse) (Malchrowicz-Mośko and Munsters, 2018). It is therefore important to study whether innovative sports events (such as dragon boat races in the case of water sports) are really needed and helpful in modern society – do they actually have an impact on positive attitudes toward physical activity, or are they just a “marketing ploy” and provide mainly only economic benefits to organizers and tourist regions (but in terms of promoting sports they are no different from standard/traditional sporting events)?

### Sports Legacy of Sports Events

There is a perception that mega-sports and other elite major events evoke euphoria among sports fans, which can translate into motivation and enthusiasm about being active (Murphy and Bauman, 2007; Bauman et al., 2009). The organizing committee of the 2012 Olympics in London promised that the Games would contribute to the promotion of sports participation among all social groups in Great Britain (Girginov and Hills, 2008). Promotion of sport – both Olympic sports and mass sports - is the alleged (mostly political) benefit of elite sporting events, but according to researchers (Weed et al., 2015), the facts do not fully confirm this claim, and effective funding to ensure increasing participation in events has yet to be developed (Chalip et al., 2017). While the promotion of sport and physical activity has long been considered a key element of the Olympic legacy, efforts to evaluate results have proved difficult, and legacy assessment methods are not standardized in all host cities (Weed et al., 2008). Researchers do not find any empirical evidence to suggest so-called sports inspiration, demonstration effect, or trickle-down effect from elite competitions in the development of mass sport. Despite their success at important events, elite athletes do not seem to inspire amateurs to do sports independently (Misener et al., 2015; Taks et al., 2018). Results obtained from previous studies show that elite sport can have a positive impact on the promotion of physical activity, although this applies more to people who already do sport – those who already participate in sport can be motivated even more in a given discipline or try other sports, but people who do not participate in pre-event sports do not seem to be encouraged to participate in sports on a daily basis (Weed et al., 2015; Storm et al., 2018). Studies show the existence of a demonstration effect for people already involved in sport, but not for new participation in sport (Taks et al., 2014). For example, it has been shown that the desire to participate in the marathon in Geneva was for participants one of the main reasons for increasing training in running (Hautbois et al., 2019). For their part, Storm and Holum (2020) did not find any direct positive effects from local sporting success on mass sport participation (Storm and Holum, 2020). Some studies show that observing a mass event has a positive impact on the willingness to engage in regular physical activity as well as the willingness to take part in a sporting event in the future, but such studies have tended to be declarative in nature (Malchrowicz-Mośko et al., 2019b). However, Taks et al. (2018) argued in favor of other positive effects that may translate into increased interest in sport in the future (so-called long-term effects), including increasing alliances between sports organizations, event organizers, and non-sport stakeholders (to strengthen the link between events for viewers/spectators and participation in sport).

### Cultural Diffusion in Sport

We are currently observing the dynamic development of mass sports events around the world – changes are taking place not only on a quantitative level but also on a qualitative level – and many events or disciplines are modified and modernized to attract as many people as possible, including sports tourists. The market for mass sports events targeting adults is already very saturated nowadays, and more and more initiatives are also targeting young people. For this purpose, some sporting events focus on the competitive element of competition, with extreme elements or cultural novelty being added. An example would be horse boarding competitions in winter in the Podhale region in Poland, practiced during an event that previously consisted mainly of racing in traditional *kumoterki* (Malchrowicz-Mośko and Munsters, 2018), or dragon boat competitions that are spreading fast around the world.

Dragon boat competitions, originating in China and organized in many countries today, are an example of cultural diffusion in sport. As Kantor noted, until recently, cultural diffusion most often required direct contact between cultures, i.e., between carriers of different cultural elements (for example, between local athletes and sports tourists). Today, this process is also mediated by the media, in particular television and the internet, whereby personal contact is no longer necessary (Kantor, 2017). One example of cultural diffusion in sport may be the international spread of cricket – a sport traditionally originating from England, which is now popular in most countries that have close ties to it. In this case, the elite tried to actively promote this sport for hegemonic purposes (Kaufman and Patterson, 2005).

The International Olympic Committee plays a particularly important role in the process involving sport and sporting events promotion. The Committee strives to promote mass sport within the widest possible social circles, but most often by promoting Western sports. Pfister believes that such westernization of physical culture can harm traditional sports in non-Western cultures of the world (Pfister, 1997). Excessive promotion of the Western concept of sport in the spirit of the ideas put forward by Citius, Altius, and Fortius may contribute to the disappearance of traditional forms of physical activity. Kabzińska-Stawarz cites Asian sports culture as an example. The researcher notes that we are observing a retreat from traditional sports, characteristic of the peoples of Asia, due to activities aimed at popularizing disciplines of foreign origin among Asians. They are popular among Asian youth because Western sports provide them with a substitute for the modern world. As an example of such actions, Kabzińska-Stawarz refers to the South Korean Society “Sport for All,” which, in parallel with calls for active participation in sport, expanded Korean facilities for mass jogging, swimming, tennis, and badminton. Among the disciplines present in Korean culture for centuries, activists of the Society noticed only archery, also granting its significance for the physical development of the population. However, when it comes to Asian combat sports, their continued survival in Asia, according to ethnologists, is at stake. Combat practices are dying out because there are no conditions for their development, and often a willingness to embrace Western fashion carries with it a need for European sports and attractions that young Japanese, Chinese, or Korean citizens find fascinating. Their drive toward modernity is as great as Western fascination with Eastern tradition. To survive, today's masters often emigrate, sell their soul and body to cinematography in their search for western sponsors, try to modernize their practices, and offer them in a hitherto unknown attractive form and taste (Kabzińska-Stawarz, 1991). Wagner believes that the promotion of similar sports activities in different countries will lead to cultural diffusion in sport which will create a global sport culture (Wagner, 1990).

### Theory of Planned Behavior in Physical Activity

One of the most important psychological theories in contemporary health and exercise psychology is the Theory of Planned Behavior. The Theory of Planned Behavior is a social cognitive theory that has guided much research on physical activity. This theory specifies that some or all of the four main psychological variables influence human behavior (Neipp et al., 2013; The Theory of Planned Behavior, 2020): attitude, intention, perceived behavioral control, and subjective norm. Intention to pursue a type of behavior is the key determinant of whether or not an individual engages in that behavior. Intention is reflected in willingness and how much effort that individual is planning to exert to pursue that behavior. The stronger the intention, the more likely an individual will engage in that behavior. Attitude represents positive or negative evaluation when pursuing a type of behavior, while subjective norm reflects the perceived social pressure that individuals feel when pursuing a type of behavior or not. For its part, perceived behavioral control represents the perceived ease or difficulty of pursuing a type of behavior.

In our work, we decided to look into attitudes toward physical activity before participating in a sports event on the water, what participants' motivations were behind participation, and how participation in a water sports event affected attitudes toward physical activity after the event. We wanted to ascertain whether the type of event (modern vs. traditional) can affect participants' attitudes and motivation, and also examined gender-related motivation.

### Aims of the Study and Research Questions

The aim of the study was to identify relationships between attitudes toward physical activity, participation in water sports, and recreational events of a modern and traditional nature. We were interested in the attitudes of participants of both types of events before participating in competitions, and whether modern water sports and recreational events (e.g., dragon boat competitions) were more effective than traditional events in promoting active lifestyles after competing, in the opinion of respondents. Therefore, the main research question was: does our society need any modern sporting events when it comes to promoting a healthy lifestyle and sport, or are these events just a marketing trap and provide mainly economic benefits to organizers of sporting events and the tourist regions in which they take place? Moreover, we decided to examine participants' motivation behind modern and traditional water events.

In our task, we asked ourselves detailed research questions as follows:

Q1: Whether modern water events are more effective than traditional water events in attracting physically inactive people – is there a statistically significant relationship between the lifestyle and the type of sports event chosen (modern vs. traditional)?
Q2: Do modern water events attract people who have no experience with water sports?
Q3: Which type of water event promotes an active lifestyle more?
Q4: Which type of water event could most encourage people to take part in the same competitions in the future?
Q5: Which type of water event provides the most positive experiences?
Q6: Which type of water event encourages people to willing practice water sports regularly?
Q7: Whether gender, age, or education influence the type of water sports event chosen?
Q8: What are the social, emotional, body-related, sport-related, and health-related motives for participation in water sports events?
Q9: What are the gender-related motives for participation in modern and traditional water events?

## Materials and Methods

### Measurements

We used a diagnostic survey method during four water sports and recreational events that took place in 2019 in the Poznan agglomeration in Poland (two traditional and two modern). Athletes who did not take part in the event were excluded. In total, 394 respondents - participants in the events - took part in the survey. The following research tool was used: a survey questionnaire examining the attitudes of active participants toward physical activity/lifestyle shown by the respondents before participating in the event and the declared impact of the event on attitudes toward physical activity after the event. Surveys were completed in writing by amateur athletes during the event, and the selection of motives for participation analyzed in the event was based on the MPAM-R (Motives for Physical Activity Measure - Revised) scale. MPAM-R refers to the motives for people practicing sports. The following aspects are assessed; pleasure (e.g., “*It's fun”*), physical fitness (e.g., “*I want to be physically fit”*), competence (e.g., “*I like engaging in physically challenging activities”*), social relations (e.g., “*I want to be with my friends”*), and appearance (e.g., “*I want to maintain my weight so I look better”*). Pleasure refers to physical activity simply because it is interesting, fun, pleasant, and makes participants happy. Physical fitness refers to being healthy/physically active, energetic, and strong. Challenge/competence means being physically active due to the need to improve during activity, acquire new skills, and face a challenge. Social relations means being with friends and meeting new people during sport activities. Appearance refers to increasing physical attractiveness, better appearance, and achieving or maintaining the desired weight. The Cronbach's alpha coefficient obtained was 0.90.

MPAM-R is a revised version of an earlier scale with the same name. The previous measurement by Frederick and Ryan (1993) was shorter (three motivations). The longer version was elaborated by Ryan, Frederick, Lepes, Rubio, and Sheldon (Frederick and Ryan, 1993; Ryan et al., 1997). A 7-point Likert scale was used.

### Procedure

All participants were treated according to the ethical guidelines of the American Psychological Association regarding the consent, confidentiality, and anonymity of the participants. All the terms appearing in the survey were explained to the respondents – for example, they contained information on current World Health Organization guidelines regarding the recommended level of physical activity and what is recognized by WHO as an active and passive lifestyle. The organizer's written consent was given to conduct the study, and participants were informed about the significance of the study and kindly requested to provide information. The survey was anonymous, voluntary, and confidential.

### Data Analyses

Efforts were made to choose a research sample in such a way as to ensure the best representativeness of the results obtained. Information from the organizers on the expected number of participants was used to determine the number of respondents. The calculation used the sample size formula for the finite population, and it was assumed that maximum estimation error (e) at the 95% confidence level should not exceed 4%.

Descriptive statistics were calculated to characterize the test group and its parameters (plus the means for all parameters were recalculated), with statistical significance set at *p* < 0.05. The groups were compared with each other using Student *t*-tests for two independent samples – it was ascertained whether the motives for both groups differed significantly. For its part, Levene's test was used to ascertain whether the variances in both groups for the trait subject were homogeneous, or whether it was necessary to correct the variance heterogeneity in the *t*-test. The chi-square test was used for comparisons in the case of qualitative features. The last method/group of methods used was cluster analysis, which made it possible to distinguish between groups of people characterized by similar attitudes in the motives subject– k-means cluster analysis and the Ward method were used for this purpose. This allowed for the creation of groups with similar motives for participating in events. In the case of measuring motivation depending on gender, Student *t*-tests for independent samples were also used and, where necessary, a correction for non-uniform variance was applied (0.55: no differences, 0.001: statistically significant differences).

In order to conduct empirical research, sport events in the Poznan agglomeration selected for analysis were divided into modern and traditional water events. Statistical analyses were performed using the Statistical 10.0 program (Statsoft; Inc., 2011, Cracow, Poland).

### Modern Events

Poznan Dragons – competition in dragon boats. The competition aims to promote water sport disciplines through fun and rivalry and to offer an attractive, new, and active way to spend free time with friends. Dragon boats, deriving from the Chinese tradition, constitute a modern alternative for Polish society (although in China these competitions are very traditional and have a long history from the Poles' standpoint, such an event available to amateurs is a new, modern activity for Polish athletes, and due to cultural diffusion has only reached Poland relatively recently).

Poznan Canoe Challenge – is a competition involving tourist canoeing, geared toward amateurs. Participants have a distance of 5 km to cover. These two modern events involving rivalry, carried out in the form of a family picnic, are aimed at promoting new forms of celebrating physical culture in the urban environment.

### Traditional Events

Another physical activity during which empirical research was conducted are two traditional recreational kayaking trips: river rafting for the residents of the Poznan agglomeration (organized in Luboń) and river rafting under the name 8. Edition of the Canoe “Critical Mass.”

### Characteristics of Respondents

A total of 394 participants of water sport and recreational events took part in the study, and below is a metric that demonstrates the socio-demographic profile of these respondents.

#### Poznan Canoe Challenge *n* = 82

Gender: female – 30 (36.6%), male – 52 (63.4%); Age: 18 or under – 0 (0%), 19–30 – 39 (47.6%), 31–40 – 22 (26.8%), 41–50 – 13 (15.8%), 51 or over – 8 (9.8%); place of residence: Poznan – 52 (63.4%), Great Poland region (<30 km from Poznan) – 21 (25,6%), Great Poland region (more than 30 km from Poznan) – 4 (4.9%), outside Great Poland region – 5 (6.1%); population of the place of residence: village – 17 (20.7%), city with <10,000 inhabitants – 2 (2.4%), city with 10,000–100,000 inhabitants – 7 (8.5%), city with 100,000–500,000 inhabitants – 4 (4.9%), city with more than 500,000 inhabitants – 52 (63.4%); level of education attained: Primary – 0 (0%), Secondary – 36 (43.8%), vocational – 3 (3.6%), incomplete higher education – 9 (11%), completed higher education – 34 (41.5%); current employment status: school pupil – 2 (2.4%), university student – 7 (8.5%), professionally active – 70 (85.3%), unemployed – 1 (1.2%), pensioner – 2 (2.4%).

Poznan Canoe Challenge participants were most often young men (19–30 years old) living in Poznan, professionally active, and had completed higher or secondary education.

#### Poznan Dragons (Dragon Boat Races) *n* = 100

Gender: female – 47 (47%), male – 53 (53%); age: 18 or under – 1 (1%), 19–30 – 39 (24%), 31–40 – 25 (30%), 41–50 – 22 (34%), 51 or over – 13 (11%); place of residence: Poznan – 61 (61%), Great Poland region (<30 km from Poznan) – 21 (21%), Great Poland region (more than 30 km from Poznan) – 14 (14%), outside Great Poland region – 4 (4%); population of the place of residence: village – 10 (10%), city with <10,000 inhabitants – 6 (6%), city with 10,000–100,000 inhabitants – 10 (10%), city with 100,000–500,000 inhabitants – 12 (12%), city with more than 500,000 inhabitants – 62 (62%); level of education attained: Primary – 3 (3%), Secondary – 35 (35%), vocational – 3 (3%), incomplete higher education – 11 (11%), completed higher education – 48 (48%); current employment status: school pupil – 1 (1%), university student – 16 (16%), professionally active – 67 (67%), unemployed – 8 (8%), pensioner – 8 (8%).

Participants in Poznan Dragons (dragon boat competitions) were most often men, aged 41–50, from Poznan, had completed higher education, and were professionally active.

#### Traditional Canoeing Rafting in Luboń *n* = 100

Gender: female – 47 (47%), male – 53 (53%); age: 18 or under – 1 (1%), 19–30 – 24 (24%), 31–40 – 30 (30%), 41–50 – 34 (34%), 51 or over – 11 (11%); place of residence: Great Poland region (<30 km from Poznan) – 100 (100%); population of the place of residence: city with 10,000–100,000 inhabitants – 100 (100%); level of education attained: Primary – 0 (0%), Secondary – 22 (22%), vocational – 10 (10%), incomplete higher education – 8 (8%), completed higher education – 60 (60%); current employment status: school pupil – 0 (0%), university student – 13 (13%), professionally active – 82 (82%), unemployed – 5 (5%), pensioner – 0 (0%).

Participants in traditional canoe rafting were most often men aged 41–50 who had completed higher education (from the city of Luboń).

#### Canoe “Critical Mass” *n* = 112

Gender: female – 52 (46.4%), male – 60 (53.6%); Age: 18 or under – 1 (1%), 19–30 – 27 (27%), 31–40 – 48 (42.9%), 41–50 – 24 (21.4%), 51 or over – 7 (6.2%); place of residence: Poznan – 78 (69.9%), Great Poland region (<30 km from Poznan) – 26 (23.2%), Great Poland region (more than 30 km from Poznan) – 6 (5.4%), outside Great Poland region – 2 (1.8%); level of education attained: Primary – 3 (2.7%), Secondary – 23 (20.5%); vocational – 0 (0%), incomplete higher education – 0 (0%), completed higher education – 86 (76.8%); current employment status: school pupil – 5 (4.5%), university student – 9 (8%), professionally active – 95 (84.8%), unemployed – 3 (2.7%), pensioner – 0 (0%).

Participants in the Poznan Critical Mass kayaking event were most often men, aged 31–40, and professionally active.

Summary basic information about the participants of the survey was divided into modern and traditional events.

##### Modern Water Events in Polish Society

Poznan Canoe Challenge + Dragon Boats *N* = 182 (most often men aged 19–30): gender: female – 77 (42%), male: 105 (58%); age: 18 years or under – 1 (0.55%), 19–30 – 78 (42.86%), 31–40 – 47 (25.82%), 41–50 – 35 (19.23%), 51 or over – 21 (11.54%).

#### Traditional Water Events in Polish Society

Canoeing rafting + Canoe Critical Mass *N* = 112 (most often men aged 31–40): gender: female – 99 (46.7%), male – 113 (53.3%); age: 18 or under – (3.3%), 19–30 – 51 (24.06%), 31–40 – 78 (36.79%), 41–50 – 58 (27.36%), 51 or over – 18 (8.49%).

## Results

Fourteen research hypotheses were put forward, and their verification and study results are provided below. Tables 1–9 show the results related to hypotheses 1–9.

**Table 1:** Lifestyle before the event.

	**Active**	**Passive**
Participants in modern events	147	35
	80.8%	19.2%
Participants in traditional events	149	63
	70.3%	29.7%

**Table 2:** Previous participation in traditional rafting.

	**Yes**	**No**
Participants in modern events	111	71
	61%	39%
Participants in traditional events	158	54
	74.5%	25.5%

**Table 3:** Impact of the event on the desire to lead an active lifestyle.

	**Average**
Modern events	5.27
Traditional events	5.13

**Table 4:** Desire to participate in the same event again.

	**Average**
Participants in modern events	5.37
Participants in traditional events	6.10

**Table 5:** Level of positive emotions during events.

	**Average**
Modern events	5.8
Traditional events	6.1

**Table 6:** Willingness to regularly practice water sports after events.

	**Average**
Participants in modern events	5.1
Participants in traditional events	5.6

**Table 7:** Gender.

	**Male**	**Female**
Participants in modern events	105	77
	57.7%	42.3%
Participants in traditional events	113	99
	53.3%	46.7%

**Table 8:** Age (young participants vs. older participants).

	**Up to 30**	**Over 40**
Participants in modern events	80	56
	58.8%	41.2%
Participants in traditional events	58	76
	43.3%	56.7%

**Table 9:** Level of education attained.

	**Higher**	**Not higher**
Participants in modern events	102	80
	56%	44%
Participants in traditional events	68	144
	32.1%	67.9%

### H.1. People Who Do Not Lead an Active Lifestyle More Often Participate in Modern Water Events Than in Traditional Water Events

This question in the survey questionnaire was aimed at ascertaining whether modern events were more effective than traditional events in attracting physically inactive people – ascertaining whether there is a statistically significant relationship between lifestyle and type of event chosen (modern vs. traditional). The chi-square test was used for this purpose, and it was ascertained whether the percentage of people leading an active/passive lifestyle differed between traditional and modern events. The test result showed that among respondents there was a statistically significant relationship between lifestyle and the event chosen – however, modern events were more often attended by physically active people (over 80% active, in traditional events: 70% active) – *p* = 0.016. Therefore, this hypothesis has not been confirmed and the reality of the situation is the opposite of that assumed. Therefore, modern sports events on water do not have any more power to attract physically inactive people than traditional water events, with more people who are not physically active choosing traditional water events – kayaking or rafting.

### H.2. People Who Have Never Participated in Canoeing or Rafting Are More Likely to Participate in Modern Water Events Than People Who Have Participated in Traditional Water Events

The aim was to see whether modern water events attract people who had no experience with these types of water sports, for which purpose the chi-square test was used. Among people who had never participated in kayaking or rafting, a modern event was more often chosen (*p* = 0.004). Almost 40% of people taking part in a modern event were taking part in kayaking for the first time. In the case of a traditional event, this was 25%, and the hypothesis was confirmed – it turned out that a modern event on water more often attracts people who have not had any experience in this sport discipline than people who chose traditional canoeing events. In a sense, modern events are therefore effective in promoting water sports in Polish society.

### H3. People Who Participate in a Modern Water Event Express Greater Willingness to Lead an Active Lifestyle Than People Who Participate in a Traditional Water Event

This question used a Likert scale (1: to a very low degree, 7: to a very great degree), by means of which it was ascertained which type of event most promotes an active lifestyle (after the event) in the respondents' declarative opinion. The *t*-test was used for two independent samples (Student *t*-test for two independent samples), and homogeneity of variance was also verified (using Levene's test). The variances proved to be homogeneous (*p* = 0.072), and so it was not necessary to take any correction related to heterogeneous variance into consideration. The difference in willingness to lead an active lifestyle did not statistically significantly differ between modern and traditional events (5.27 vs. 5.13), *p* = 0.413. Therefore, the hypothesis has not been confirmed – in the opinion of respondents, modern water events do not produce a greater desire to lead an active lifestyle (in the declarative sphere) than traditional water events.

### H4. People Who Take Part in a Traditional Water Event Are More Willing to Participate Again in the Same Water Event in the Future Than People Who Take Part in a Modern Water Event

It was ascertained which type of event could most encourage people to take part in the same competitions in the future and whether modern events were just a one-off sports experience (an impermanent “curiosity”). The Student *t*-test was used for two independent samples, and homogeneity of variance was also verified (using Levene's test). The variances proved not to be homogeneous, and so it was necessary to take the patch related to heterogeneous variance into consideration. The hypothesis has been confirmed, with the difference in willingness to take part in the same event again being significantly greater in the case of a traditional event (6.1 vs. 5.4), *p* < 0.001. Therefore, modern events, compared to traditional events, are only a one-time experience and the people surveyed more often want to return to traditional kayaking. This can be due to many factors – e.g., the possibility of contact with nature during river rafting or attachment to rafting.

### H5. There Are Greater Positive Emotions Deriving From Participation in a Modern Event Than a Traditional Event

It was assumed that participation in a new type of sport would provide more positive experiences - fun, contact with a new form of physical culture, strong sports emotions, and above all, rivalry. The *t*-test was used for two independent samples (Student *t*-test for two independent samples), and homogeneity of variance was also verified (using Levene's test). The variances proved not to be homogeneous, and so it was necessary to take the patch related to heterogeneous variance into consideration. The difference in level of positive emotions in the event is significantly greater in the case of a traditional event (6.1 vs. 5.8), *p* = 0.048. As such, the hypothesis has not been confirmed, with traditional canoeing or rafting providing participants with positive emotions at a higher level than a modern event.

### H6. Participants in Traditional Rather Than Modern Events Declare Their Greater Willingness to Regularly Practice Water Sports

The *t*-test was used for two independent samples (Student *t*-test for two independent samples), and homogeneity of variance was also verified (using Levene's test). The variances proved not to be homogeneous, and so it was necessary to take the patch related to heterogeneous variance into consideration. The difference in willingness to practice sports regularly was significantly greater in the case of a traditional event (5.6 vs. 5.1), *p* = 0.008. The hypothesis has been confirmed and, therefore, modern water events do not encourage people to practice water sports more regularly (canoeing).

### H7. Men More Often Take Part in a Modern Water Event Than in a Traditional Event

The chi-square test was used for this purpose, and there was no statistically significant relationship between gender and choice of modern or traditional event (*p* = 0.38). In a modern event, 57.7% of participants were men, whereas in a traditional event it was 53.3%, although the differences were not statistically significant. The hypothesis has thus not been confirmed.

### H8. Young People (Up to 30 Years Old) More Often Take Part in a Modern Water Event Than Older People (Over 40 Years Old)

Analyzing the two groups (under 31 and over 40), it can be seen that the difference was statistically significant – older people were more likely to choose a traditional event than younger people (in traditional events older people accounted for 56.7% of participants, whereas in a modern event young people accounted for 58.8% of respondents), *p* = 0.008. The hypothesis has thus been confirmed – modern water events are an attractive option primarily for young people under 30 years of age.

### H9. People Who Have Completed Higher Education Are More Likely to Attend a Traditional Water Event Than a Modern Water Event

It was assumed that people who have completed higher education value traditional forms of sport (they appreciate, for example, contact with nature, they are more demanding, it is more difficult to convince them to partake in new, unproven forms of leisure time spending), and with new options it is easier to reach the masses – i.e., people who have not completed higher education. The difference is statistically significant (*p* = 0.000), although the hypothesis has not been confirmed and the situation is reversed, as people who have completed higher education were statistically more likely to choose modern events (therefore there is a statistically significant relationship between having completed higher education and choosing the type of kayaking event). Modern events more often attract people who have completed higher education (perhaps also for reasons such as fashion or social prestige).

Below are the hypotheses related to the motivation behind participation:

10. Social motives are more often declared by participants in a modern event than a traditional event (Hypothesis confirmed).
11. Emotional motives are more often declared by participants in a modern event than a traditional event (The hypothesis was not confirmed; the reverse is true).
12. Body-related motives are more often declared by participants in a traditional event than a modern one (The hypothesis was not confirmed; the reverse is true).
13. Sport-related motives are more often declared by participants in a traditional event than a modern one (The hypothesis was not confirmed; the reverse is true).
14. Health-related motives are more often declared by participants in a traditional event than a modern one (The hypothesis was not confirmed; the reverse is true).

The *t*-test was used for two independent samples (Student *t*-test for two independent samples), and homogeneity of variance was also verified (using Levene's test). Answers to individual questions (detailed motives) as well as the sum of motives for individual groups were examined accordingly, and the Table 10 shows the average values for the sum of ratings of individual motives, broken down into participants taking part in traditional and modern events.

**Table 10:** Average values for the sum of ratings of individual motives, broken down into participants taking part in traditional and modern events.

**Motives**	**Event**	**Average**	***p*-value**	**Significant difference?**
Social	Modern	25.02	0.00099	Yes
	Traditional	22.91		
Emotional	Modern	38.62	0.03328	Yes
	Traditional	40.24		
Body-related	Modern	39.68	0.03195	Yes
	Traditional	37.80		
Sport-related	Modern	30.91	0.03744	Yes
	Traditional	29.72		
Health-related	Modern	30.51	0.00000	Yes
	Traditional	26.41		

The group participating in traditional canoeing events showed (in relation to the group participating in modern events) significantly greater pro-emotional motives, while in the group taking part in modern events there were significantly greater social, body-, sport-, and health-related motives. Analyzing the individual questions (without combining them into groups of motives), it can be seen that for some the differences are insignificant. The exact results are shown in the Table 11.

**Table 11:** The reasons for doing sport.

**Motives**	**Event**	***N* (respondents)**	**Average**	***p*-value**	**Statistically** ** significant difference?**
**A. Social**					
A.1*I want to be with my friends*	Modern	182	5.86	0.180	No
	Traditional	212	5.65		
A.2*I like to be with others who are interested in this sporting activity*	Modern	182	5.21	0.399	No
	Traditional	212	5.06		
A.3*I want to meet new people*	Modern	182	4.76	0.029	Yes
	Traditional	212	4.32		
A.4*My friends want me to take part*	Modern	182	3.75	0.000	Yes
	Traditional	212	2.93		
A.5*I enjoy spending time doing this sporting activity with other people*	Modern	182	5.43	0.014	Yes
	Traditional	212	4.96		
**B. Emotional**					
B.1*It's fun*	Modern	182	6.19	0.049	Yes
	Traditional	212	6.43		
B.2*I like doing this activity*	Modern	182	5.20	0.001	Yes
	Traditional	212	5.74		
B.3*It makes me happy*	Modern	182	5.29	0.000	Yes
	Traditional	212	5.96		
B.4*I think it's interesting*	Modern	182	5.58	0.270	No
	Traditional	212	5.74		
B.5*I enjoy this activity*	Modern	182	5.54	0.172	No
	Traditional	212	*5.75*		
B.6*I find this activity stimulating*	Modern	182	5.23	0.734	No
	Traditional	212	5.28		
B.7*I like the excitement of participating*	Modern	182	5.60	0.120	No
	Traditional	212	5.33		
**C. Sport-related (challenge, rivalry, etc.)**					
C.1*I like engaging in physically challenging activities*	Modern	182	5.45	0.585	No
	Traditional	212	5.35		
C.2*I want to acquire new skills*	Modern	182	5.34	0.669	No
	Traditional	212	5.42		
C.3*I want to improve my existing skills*	Modern	182	5.42	0.635	No
	Traditional	212	5.50		
C.4*I like the challenge*	Modern	182	5.94	0.003	Yes
	Traditional	212	5.46		
C.5*I want to maintain my current level of skill*	Modern	182	5.68	0.008	Yes
	Traditional	212	5.23		
C.6*I like activities which are physically challenging*	Modern	182	5.73	0.003	Yes
	Traditional	212	5.23		
C.7*I want to get better at my activity*	Modern	182	6.13	0.001	Yes
	Traditional	212	5.61		
**D. Health-related**					
D.1*I want to be physically fit*	Modern	182	6.33	0.223	No
	Traditional	212	6.17		
D.2*I want to have more energy*	Modern	182	6.19	0.124	No
	Traditional	212	5.98		
D.3*I want to improve my cardiovascular system*	Modern	182	5.95	0.005	Yes
	Traditional	212	5.50		
D.4*I want to maintain my physical strength in order to live a healthy lifestyle*	Modern	182	6.18	0.147	No
	Traditional	212	5.98		
D.5*I want to maintain my well-being and physical health*	Modern	182	6.26	0.212	No
	Traditional	212	6.09		
**E. Body-related**					
E.1*I want to maintain my weight so that I look better*	Modern	182	5.59	0.000	Yes
	Traditional	212	4.86		
E.2*I want to define my muscles so that I look better*	Modern	182	5.64	0.003	Yes
	Traditional	212	5.07		
E.3*I want to improve my appearance*	Modern	182	5.43	0.011	Yes
	Traditional	212	4.95		
E.4*I want to look attractive for others*	Modern	182	5.31	0.000	Yes
	Traditional	212	4.16		
E.5*I want to improve my body shape*	Modern	182	5.54	0.003	Yes
	Traditional	212	4.99		
E.6*I feel physically unattractive if I don't*	Modern	182	2.99	0.007	Yes
	Traditional	212	2.39		

In 13 questions, the groups participating in modern and traditional events did not differ significantly, while in the case of 17 questions, the differences in answers between the two groups were statistically significant. This translates into statistically significant differences within each of the motives analyzed (four in favor of modern events and one in favor of traditional events).

#### Cluster Analysis

It was decided that a cluster analysis would be appropriate, using the k-means method, and it was decided that the number of clusters should be set at level 5. This choice was also confirmed by creating a dendrogram (using a hierarchical cluster analysis, Ward's method). Two clusters were also verified, although the division into two clusters did not have any cognitive value. The separation of five clusters allowed five attitudes to be defined that characterize participants in water sports and recreational events when making decisions about the choice of event, and these clusters were established based on standardized response values in five motive groups.

The numbers of individual groups are shown in the Table 12.

**Table 12:** Number of individual groups are shown.

**Number of clusters**	**Number of respondents**
1	95
2	55
3	33
4	148
5	63

The membership of individual types of event for clusters is shown in the Table 13.

**Table 13:** The number of participants based on the type of sport event.

		**Cluster**					**Total**
		**1**	**2**	**3**	**4**	**5**	
Participants in traditional events	Number	62	27	20	61	42	212
	% from number of cluster observation	65.3%	49.1%	60.6%	41.2%	66.7%	53.8%
Participants in modern events	Number	33	28	13	87	21	182
	% from number of cluster observation	34.7%	50.9%	39.4%	58.8%	33.3%	46.2%
Total	Number	95	55	33	148	63	394

In groups 1, 3, and 5, participants in traditional events predominate. Focus 4 refers primarily to participants in modern events. In cluster 2, the number of participants in modern and traditional events is similar.

Using standardized data, it is possible to identify which response groups deviate from the mean (up or down). Based on this, we can also specify a participant profile.

*Cluster 1:* Low level of social motives, above average emotional, sport-, and health-related motives. They are mainly participants in traditional events. We suggest referring to them as LOVERS OF SPORTS EMOTIONS.
*Cluster 2:* Low or very low level of social, emotional, and body-related motives. These are participants in events only for health reasons – irrespective of whether it is a traditional or modern event. We suggest referring to them as HEALTHY LIFESTYLE MANAGERS.
*Cluster 3:* Very low level of any motives – a small group of people participating in the event “for punishment”, most likely at the instigation of other people. We suggest referring to them as WATER SPORTS MALCONTENTS.
*Cluster 4:* The group is very positive about all the motives. We suggest referring to them as WATER SPORTS ENTHUSIASTS.
*Cluster 5:* The group has a neutral attitude toward emotional motives and, in the case of others, a low or very low level. We suggest referring to them as NEUTRALS TO WATER SPORTS.

As we can see, two groups of respondents refer to people who probably took part in the event at the instigation of other people, one group is very enthusiastic about all motivations behind participation, one group focuses on health, and the last group focuses on sports emotions.

Group membership was also compared to “metric” data – no group stands out in terms of gender distribution (chi-square test, *p* = 0.946), age (chi-square test, *p* = 0.122), or professional situation (chi-square test, *p* = 0.068) – in the latter case it can only be noted that in cluster 3, 15% are unemployed (in the remaining cases it is <3%). As much as 20% of focus 5 contains pupils or students. However, the difference is visible in the case of education (chi-square tests, *p* = 0.000). In cluster 3, the smallest percentage refers to those people who have completed higher education (43% in total, of which 18% in cluster 3, about 50% in clusters 1 and 2, and 40% in clusters 4 and 5). In cluster 4, the largest percentage of people refer to those who have completed secondary education (31.1%), while in cluster 3, almost 40% of people have completed vocational education.

Final centers of focus (based on standardized data) are shown in Table 14.

**Table 14:** Final centers of focus.

	**Cluster**				
	**1**	**2**	**3**	**4**	**5**
Social	−0.628	−0.547	−1.070	0.961	−0.273
Emotional	0.446	−1.270	−1.489	0.501	0.038
Body-related	0.169	−0.469	−1.833	0.640	−0.388
Sport-related	0.360	0.065	−2.247	0.472	−0.532
Health-related	0.314	0.377	−0.941	0.460	−1.391

From the Table 15, we can accurately trace the average of individual questions, broken down into five groups.

**Table 15:** Average of individual questions, broken down of participants.

	**Group 1**	**Group 2**	**Group 3**	**Group 4**	**Group 5**
A.1	5.32	5.05	4.61	6.51	5.79
A.2	4.34	4.33	3.36	6.46	4.83
A.3	3.62	3.67	2.91	6.03	3.95
A.4	2.00	3.29	2.67	4.55	2.71
A.5	4.61	4.05	3.52	6.46	4.86
B.1	6.57	5.73	5.24	6.58	6.41
B.2	6.16	4.11	3.85	5.83	5.73
B.3	6.24	4.51	4.00	5.96	5.89
B.4	6.13	4.15	4.30	6.26	5.60
B.5	6.12	4.15	3.82	6.31	5.68
B.6	5.79	3.60	3.64	5.99	5.02
B.7	5.84	3.71	3.45	6.32	5.44
C.1	5.64	4.18	2.61	6.41	5.17
C.2	5.49	5.04	2.88	6.18	4.94
C.3	5.81	4.95	3.21	6.15	4.95
C.4	5.75	4.91	3.79	6.45	5.46
C.5	5.76	4.80	3.24	6.24	4.78
C.6	5.52	4.93	3.45	6.21	5.13
C.7	6.17	5.80	3.58	6.59	4.87
D.1	6.60	6.40	3.61	6.74	5.78
D.2	6.49	6.04	3.70	6.55	5.63
D.3	6.03	5.84	3.21	6.41	4.75
D.4	6.59	6.15	3.45	6.57	5.41
D.5	6.59	6.22	3.58	6.68	5.68
E.1	5.81	5.75	3.79	5.86	3.00
E.2	5.97	5.71	3.94	6.06	3.05
E.3	5.75	5.75	3.70	5.89	2.92
E.4	5.12	5.29	2.73	5.63	2.33
E.5	5.88	5.95	3.48	5.91	3.03
E.6	2.56	3.20	2.33	3.04	1.65
Social attitudes	19.88	20.40	17.06	30.01	22.14
Emotional attitudes	42.84	29.95	28.30	43.26	39.78
Body-related attitudes	40.14	34.60	22.76	44.22	35.30
Sport-related attitudes	32.31	30.64	17.55	32.94	27.25
Health-related attitudes	31.08	31.64	19.97	32.38	15.98

In the next step, we decided to ascertain gender-related motives for participation in modern and traditional water events.

No statistically significant differences were found between motivation behind men and women participating in traditional water events (Table 16).

**Table 16:** Men vs. women in traditional water events.

**Motives**	**Gender**	**Number of respondents**	**Average**	**Standard deviation**	***t* stat value**	***p*-value**
Social					−0.561	0.575
	Male	113	22.6903	5.76640		
	Female	99	23.1616	6.46925		
Emotional					−1.008	0.314
	Male	113	39.7434	8.13258		
	Female	99	40.7980	6.93406		
Body-related					0.262	0.794
	Male	113	37.9558	9.12159		
	Female	99	37.6263	9.15243		
Sport-related					0.023	0.982
	Male	113	29.7257	6.02709		
	Female	99	29.7071	5.66632		
Health-related					0.561	0.575
	Male	113	26.7345	8.98115		
	Female	99	26.0404	8.98061		

No statistically significant differences were found between the motivation behind men and women participating in modern water events (Table 17).

**Table 17:** Men vs. women in modern water events.

**Motives**	**Gender**	**Number of respondents**	**Average**	**Standard deviation**	***t* stat value**	***p*-value**
Social					−1.065	0.288
	Male	105	24.5810	6.63003		
	Female	77	25.6234	6.36818		
Emotional					−1.190	0.288
	Male	105	38.0667	7.18474		
	Female	77	39.3766	7.53595		
Body-related					−0.122	0.903
	Male	105	39.6190	8.62508		
	Female	77	39.7662	7.24550		
Sport-related					−0.283	0.777
	Male	105	30.8095	5.38176		
	Female	77	31.0390	5.41791		
Health-related					0.400	0.690
	Male	105	30.7143	7.63559		
	Female	77	30.2208	8.98482		

Statistically significant differences were found between the motivation behind men to participate in traditional and modern water events: social and health-related motives proved to be more important for men participating in modern water events (Table 18).

**Table 18:** Traditional events vs. modern events – men.

**Motives**	**Event**	**Number of respondents**	**Average**	**Standard deviation**	***t* stat value**	***p*-value**
Social					−2.251	0.025
	Traditional	113	22.6903	5.76640		
	Modern	105	24.5810	6.63003		
Emotional					1.608	0.109
	Traditional	113	39.7434	8.13258		
	Modern	105	38.0667	7.18474		
Body-related					−1.381	0.169
	Traditional	113	37.9558	9.12159		
	Modern	105	39.6190	8.62508		
Sport-related					−1.397	0.164
	Traditional	113	29.7257	6.02709		
	Modern	105	30.8095	5.38176		
Health-related					−3.512	0.001
	Traditional	113	26.7345	8.98115		
	Modern	105	30.7143	7.63559		

Statistically significant differences were also found between the motivation behind women participating in traditional and modern water events: social and health-related motives proved to be more important for women participating in modern water events (Table 19).

**Table 19:** Traditional events vs. modern events – women.

**Motives**	**Event**	**Number of respondents**	**Average**	**Standard deviation**	***t* stat value**	***p*-value**
Social					−2.522	0.013
	Traditional	99	23.1616	6.46925		
	Modern	77	25.6234	6.36818		
Emotional					1.299	0.196
	Traditional	99	40.7980	6.93406		
	Modern	77	39.3766	7.53595		
Body-related					−1.682	0.094
	Traditional	99	37.6263	9.15243		
	Modern	77	39.7662	7.24550		
Sport-related					−1.577	0.117
	Traditional	99	29.7071	5.66632		
	Modern	77	31.0390	5.41791		
Health-related					−3.063	0.003
	Traditional	99	26.0404	8.98061		
	Modern	77	30.2208	8.98482		

## Discussion

The research results provided in this article expand on the current state of knowledge about mass participation in sport – the impact of sporting events on the promotion of physical activity - and offer the motivation behind participation in modern and traditional water sports. As a result of the analysis, the following associations were found: There is a statistically significant relationship between lifestyle and the event chosen – people who were previously physically active more often participate in modern events. Modern water sports events do not have any more power to attract physically inactive people than traditional water sports events. More people who are not physically active have chosen traditional water events (kayaking). However, modern events on water are more likely to attract people who have never had any experience with canoeing than people who have chosen traditional canoeing. These results are in line with the results obtained by Bunnig and Walker (Buning and Walker, 2016), who demonstrated the importance of the contextual characteristics of sporting events. It is therefore a positive observation that modern sporting events attract people who previously had no experience with canoeing. From a practical point of view, it is worth considering how to use this potential in the future to encourage participants to practice water sports regularly.

On the other hand, no statistically significant differences were found in the respondents' willingness to lead an active lifestyle after a modern and traditional event. Modern events are therefore not more effective than traditional events (but neither are they less effective). In this case the results did not show the contextual influences previously found (Buning and Walker, 2016). Furthermore, the difference in willingness to take part in the event again is significantly greater in the case of a traditional event. It was also traditional kayaking or rafting that provided participants with greater positive emotions. The difference in willingness to practice regular water sports is also significantly greater in traditional events. Modern water events therefore do not encourage people to do water sports more regularly. From these results, we can interpret that traditional water sports are of great importance in promoting physical activity, and that added to modern sporting events, they can be an important source of promotion for regular physical activity (Crofts et al., 2012). It would seem to us that similar tendencies will be observed in studies that analyze the differences between modern and traditional events in other sports disciplines.

As for socio-demographic issues, there was no statistically significant relationship between gender and the choice of a modern or traditional event (on the other hand, almost the same participation of women in the events subject to survey was positive because, for example, in marathons the percentage of women is usually lower than men). However, as far as age was concerned, modern water events were more appealing for young people under 30 years of age than for older people (who often choose traditional kayaking). There was also a statistically significant relationship between having a university degree and choosing the type of canoeing event – modern sporting events on water more often attracted people who have completed higher education.

When it comes to motives for participation in water sports events, the following phenomena can be noted: The group taking part in traditional canoeing events showed (compared to the group taking part in modern events) significantly greater pro-emotional motives, and in the group taking part in modern events there were significantly greater pro-social, body, pro-sport, and pro-health motives. The nature of the event (modern vs. traditional) therefore significantly differentiates between the motivation behind water sports enthusiasts. Most often, modern events attract young people who have completed higher education. A cluster analysis showed, however, that it is possible to distinguish between five clear attitudes of participants: enthusiasts of sports emotions, managers of healthy lifestyles, enthusiasts of water sports (in every motivational aspect), and also malcontents and people who focused on a given event in a neutral way. Interestingly, some similar types were also observed in other sports - for example, Ogles and Masters (2003) distinguished running enthusiasts and lifestyle managers among marathon runners (however, there were also groups of people who focused on competition and achieving their own goals). Hautbois et al. (2019) also distinguished, among runners, people who did not show any strong motivation before the running event (as in the case of the above “malcontents”), which they referred to as “happy loafers” (Hautbois et al., 2019). In our paper, we also ascertained the gender-related motives for participation in modern and traditional water events, and social and health-related motives proved to be more important for women and men who participate in modern water events. According to a similar study conducted at a mass cycling event (Bike Challenge), the desire to be physically active was much more important for men than for women cyclists (Malchrowicz-Mośko et al., 2019a).

Our study showed that participants in water events feel encouraged to lead an active lifestyle on level 5.27 and 5.13 on a 7-point Likert scale. This information shows to what extent mass recreational events on water promote an active lifestyle. A similar study was carried out on recreational runs for amateurs, e.g., Park Run – participants rated the level of encouragement at 4.99 on a 7-point Liker scale (Malchrowicz-Mośko et al., 2020b), while in the case of water events for amateurs, this was assessed at a slightly higher level. It is therefore deemed worthwhile for water sports events to become as popular as mass running events, as they promote active lifestyle to the same degree among amateurs.

## Conclusions

The analysis shows that modern sports events on water are no more effective in promoting an active lifestyle than traditional events (rafting). However, people who had previously had no contact with canoeing and were more encouraged to practice water sports were in favor of modern sports events on water compared to traditional events. This is a positive observation, and the test results can prove useful for people involved in sport management and the promotion of healthy habits and active lifestyles. From a practical point of view, it is worth considering how this potential may be used in the future to encourage participants to practice water sports regularly.

Our study also provides knowledge for sport and public health managers about the motivational profiles of participants in water sports events, crucial health and social aspects of the participants, and how motivation behind participation is shaped by gender.

The key strength of the study is the focus on water sports disciplines and their links with attitudes toward physical activity. The limitation of the study lies in the declarative nature of the research – in the future it would be worth analyzing the lifestyle of participants sometime after the end of the event. It would be also worth ascertaining how the participants' motivation behind traditional and modern water sports events are shaped due to specific socio-demographic issues, e.g., age and marital status.

## Data Availability Statement

The raw data supporting the conclusions of this article will be made available by the authors, without undue reservation.

## Ethics Statement

The research was carried out in accordance with the Declaration of Helsinki, and the study was treated in accordance with the guidelines of the Publication Manual of the American Psychological. Written informed consent to participate in this study was provided by the participants' legal guardian/next of kin.

## Author Contributions

MM, MK, and EM-M contributed to the conception and design of the study. MM organized the database. MM and EM-M performed the statistical analysis and wrote the first draft of the manuscript. MM, EM-M, MK, PL-G, and MT-S wrote sections of the manuscript. All authors contributed to manuscript revision, read, and approved the submitted version.

## Conflict of Interest

The authors declare that the research was conducted in the absence of any commercial or financial relationships that could be construed as a potential conflict of interest.
